# Silencing of *CPSF7* inhibits the proliferation, migration, and invasion of lung adenocarcinoma cells by blocking the AKT/mTOR signaling pathway

**DOI:** 10.1515/med-2022-0570

**Published:** 2022-10-21

**Authors:** Weishao An, Fang Yu

**Affiliations:** Department of Respiratory, Xiaoshan First People’s Hospital, Hangzhou, 311200, China; Department of Respiratory, Xiaoshan First People’s Hospital, No. 199 Shixin South Road, Hangzhou, 311200, China

**Keywords:** *CPSF7*, lung adenocarcinoma, AKT/mTOR signaling pathway, antitumor

## Abstract

Cleavage and polyadenylation specific factor 7 (*CPSF7*) is an important participator in the cleavage and polyadenylation of pre-mRNAs. This study aims to uncover the function and underlying mechanism of *CPSF7* in lung adenocarcinoma (LUAD). *CPSF7* expression in LUAD cells was measured using real time-quantitative polymerase chain reaction and Western blotting. Our results showed that CPSF7 expression was upregulated in LUAD cell lines (A549, H1299, and HCC827). To explore the function of *CPSF7* on LUAD, *CPSF7* was silenced by the si-*CPSF7* transfection and overexpressed by the oe-*CPSF7* transfection in A549 cells. Cell proliferation was measured using cell counting kit-8 and colony formation assays. Cell migration and invasion were measured by wound healing and Transwell assays, respectively. Our data revealed that *CPSF7* silencing inhibited the viability, colony formation, migration, and invasion of LUAD cells. On the contrary, *CPSF7* overexpression enhanced the malignant characteristics of LUAD cells. Additionally, expression of AKT/mTOR pathway-related proteins was detected using Western blotting. *CPSF7* silencing blocked the AKT/mTOR signaling pathway. The intervention of SC79 (an activator of the AKT/mTOR pathway) weakened the antitumor effects of *CPSF7* silencing in LUAD cells. Silencing of *CPSF7* inhibits the malignant characteristics of LUAD cells by blocking the AKT/mTOR signaling pathway.

## 1 Introduction

Lung adenocarcinoma (LUAD) is an aggressive and fatal tumor that originates from small airway epithelial or type II alveolar cells [1]. As the most common histological subtype, LUAD accounts for about 40% of all lung cancers [2]. Until now, LUAD remains one of the leading causes of cancer-related death globally despite the advances in understanding the pathogenesis and developing novel therapeutic strategies [3,4]. The disseminated metastatic tendency and chemoradiotherapy resistance are still the major challenges to therapeutic effectiveness [4,5]. With the development of molecular targeted therapy, the discovery of novel targets with high efficiency is urgently needed.

Alternative polyadenylation (APA) is a necessary processing step for the maturation of eukaryotic mRNAs, and its abnormality contributes to diverse oncological, immunological, neurological, and hematological disorders [6]. Cleavage and polyadenylation specific factor (*CPSF*) is one of four key protein complexes in APA [7]. Previous studies have determined that CPSFs play important roles in the tumorigenesis and progression of different types of cancers. For example, the upregulation of *CPSF1* in hepatocellular carcinoma (HCC) tissues is correlated with poor survival outcomes, and *CPSF1* knockdown inhibits the proliferation and migration of HCC cells *in vitro* [8]. *CPSF4* is upregulated in colorectal cancer tissues, and its knockdown inhibits the proliferation, migration, invasion, and stemness maintenance of colorectal cancer cells *in vitro* [9]. In addition, the upregulation of *CPSF4* is also correlated with the poor overall survival of patients with LUAD [10]. Knockdown of *CPSF4* can inhibit the proliferation, migration, and invasion of lung cancer cells *in vitro*, as well as the tumor growth in mice [11]. *CPSF7*, also known as *CFIm59* is a large subunit of cleavage factor involved in the cleavage and polyadenylation of pre-mRNAs (7). Fang et al. have shown that *CPSF7* is upregulated in HCC cells and its knockdown inhibits cell proliferation, colony formation, and migration [12]. Yang et al. have found that *LINC00958* knockdown inhibits the proliferation, migration, and invasion of LUAD cells via regulating *miR-625-5p/CPSF7* axis [13]. However, the specific function of *CPSF7* in LUAD and the underlying regulatory mechanisms are not fully revealed.

PI3K/AKT/mTOR pathway is a classical signaling pathway that is crucial in the regulation of basic intracellular functions, such as cell proliferation, metabolism, and motility [14]. The abnormal activation of the PI3K/AKT/mTOR pathway contributes to the malignant characteristic of cancer cells, including acquired autonomic growth signal, apoptosis resistance, angiogenesis, metastasis enhancement, and anti-growth signal insensitivity [15]. Since inhibition of the PI3K/AKT/mTOR pathway exhibits great antitumor effects against lung cancer, a variety of pan-PI3K inhibitors, selective PI3K inhibitors, AKT inhibitors, mTOR inhibitors, and dual PI3K-mTOR inhibitors have been developed in clinical trials [15,16]. In addition, the silencing of some CPSFs has also been determined to inhibit cancer progression by inhibiting the PI3K/AKT/mTOR pathway, such as the CPSF3-PI3K/Akt/GSK-3β in HCC [17], CPSF4-PI3K/AKT in LUAD [10], and CPSF7-PTEN/AKT in HCC [12]. Nevertheless, whether the regulatory role of *CPSF7* in LUAD is mediated by the AKT/mTOR pathway remains unclear.

In this study, the function of *CPSF7* in LUAD cells was evaluated in the aspects of cell viability, colony formation, migration, and invasion. The action mechanism of *CPSF7* involving the AKT/mTOR signaling pathway was further analyzed. This study is aimed to uncover a novel molecular target for the treatment of LUAD.

## 2 Materials and methods

### 2.1 Cell culture and treatment

Three human LUAD cell lines (A549, H1299, and HCC827) and one normal lung epithelial cell line (BEAS‐2B) were purchased from American Type Culture Collection (Manassas, VA, USA). Cells were cultured in Dulbecco’s modified Eagle medium (DMEM) supplied with 10% fetal bovine serum (FBS) and 1% penicillin/streptomycin at 37°C with 5% CO_2_. The siRNA targeting *CPSF7* (si-*CPSF7*), overexpression vector carrying *CPSF7* (oe-*CPSF7*), and corresponding negative controls (si-NC and oe-NC) were purchased from RiboBio (Guangzhou, China). The above-mentioned vectors were packaged in lentivirus and then transfected into A549 cells using Highgene transfection reagent (ABclonal, Wuhan, China). In addition, the si-*CPSF7-*transfected A549 cells were further treated with 8 µg/mL SC79 (an activator of the AKT/mTOR pathway). A549 cells without treatments were considered as the controls.

### 2.2 Real time-quantitative polymerase chain reaction (RT-qPCR)

Total RNA was extracted from cells using TRIzol reagent (Invitrogen, CA, USA) and was reverse-transcribed using FastKing First-strand cDNA Synthesis Mix (Tiangen, China). RT-qPCR was performed using SYBR Green qPCR Kit (Lifeint, Xiamen, China) on Mx3000P system (Stratagene, Carlsbad, CA, USA). The RT-qPCR program was an initiative of 95°C for 3 min, followed by 40 cycles of 95°C for 15 s and 62°C for 40 s. GAPDH was used as the internal control, and relative mRNA expression of *CPSF7* was calculated by the 2^−∆∆Ct^ method. The primers used in RT-qPCR included *CPSF7*-F, 5′–GCT GAC GAG GAG TTC AAC CA–3′, *CPSF7*-R, 5′–ACG GCA GCT CGT CTA TTA CG–3′; GAPDH-F, 5′–ACT CAC GGC AAA TTC AAC GG–3′, and GAPDH-R, 5′–AGT TGG GAT AGG GCC TCT CTT G–3′.

### 2.3 Western blotting

Total proteins were extracted from cells using RIPA Lysate (Beyotime, Beijing, China). Proteins were separated using 10% SDS polyacrylamide gel electrophoresis and transferred onto polyvinylidene difluoride membranes. Subsequently, membranes were blocked with 5% nonfat milk for 1 h, incubated with primary antibody (anti-CPSF7, anti-AKT, anti-mTOR, and anti-GAPDH, 1:1,000, Abcam, Cambridge, UK; anti-p-AKT and anti-p-mTOR, 1:1,000, Cell Signaling Technology, Danvers, MA, USA) for 12 h at 4°C and further with secondary antibody (HRP-conjugated goat anti-rabbit IgG, 1:2,000, Abcam) for 1 h at 25°C. Blots were finally visualized using an efficient chemiluminescence kit (Pierce, Rockford, IL, USA) and captured under Gel Imaging System (3500, Tanon, China).

### 2.4 Cell viability assay

The viability of A549 cells was measured using cell counting kit-8 (CCK-8, Beyotime). The transfected cells were resuspended into 2 × 10^4^ cells/mL and then seeded into 96-well plates at a volume of 100 µL. After 24, 48, 72, and 96 h of culture, cells were incubated with 10 µL CCK-8 for 2 h. The optical density at 450 nm was detected using a microplate reader (DR-3518G, Hiwell Diatek, Wuxi, China).

### 2.5 Colony formation assay

The proliferation of A549 cells was evaluated using colony formation assay. The transfected cells (200 cells/well) were seeded into six-well plates and cultured for 7 days. After being fixed with 4% paraformaldehyde for 15 min and stained with 0.1% crystal violet for 20 min at 25℃, the stained colonies were captured and counted under a microscope (BX53M, Olympus, Japan).

### 2.6 Cell migration assay

The migration of A549 cells was evaluated using wound healing assay. The transfected cells were seeded into six-well plates at a density of 5 × 10^5^ cells/well and cultured overnight. A wound was then scratched using a pipette tip on each well. After being washed with PBS, cells were cultured in a serum-free medium for 24 h. The wound distance was measured under a microscope (BX53M, Olympus) before and after wounding (0 and 24 h).

### 2.7 Cell invasion assay

The invasion of A549 cells was detected using Transwell chambers. The transfected cells were resuspended in the serum-free medium into 1 × 10^6^/mL and added into a Matrigel-coated upper chamber at a volume of 200 µL. The lower chamber was added with DMEM supplied with 10% FBS. After 24 of culture, cells in the lower chamber were fixed with 4% paraformaldehyde for 30 min and stained with 0.1% crystal violet for 30 min. The stained cells were captured and counted under a microscope (BX53M, Olympus).

### 2.8 Statistical analysis

Statistical analysis was performed using GraphPad Prism 7.0 (GraphPad, San Diego, CA, USA). Data were expressed as mean ± standard deviation. The differences among multiple groups were analyzed using one/two-way ANOVA followed by Tukey’s test. *P* < 0.05 was considered statistically significant.

## 3 Results

### 3.1 The expression of *CPSF7* was increased in LUAD cells

**Figure 1:**
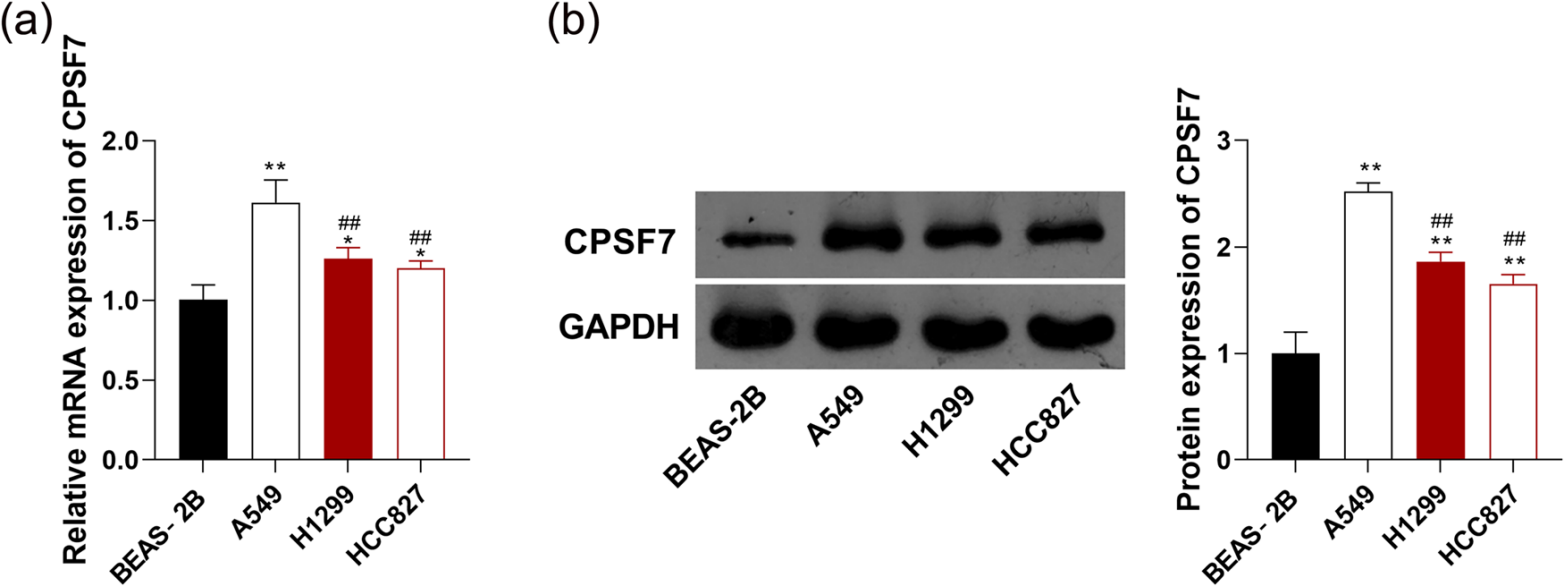
The expression of *CPSF7* in three LUAD cell lines (A549, H1299, and HCC827) and a normal lung epithelial cell line (BEAS‐2B). (a) mRNA expression of *CPSF7* was measured by RT-qPCR; (b) protein expression of CPSF7 was measured by Western blotting. Each experiment was repeated three times (*n* = 3). ^*^ *P* < 0.05; ^**^ *P* < 0.01 vs BEAS-2B; ^##^ *P* < 0.01 vs A549.

The expression of *CPSF7* was detected in LUAD cells. RT-qPCR showed that the mRNA expression of *CPSF7* was significantly higher in LUAD cell lines (A549, H1299, and HCC827) than that in normal lung epithelial cell line (BEAS‐2B) (*P* < 0.05, Figure 1a). Western blotting also determined significantly higher protein expression of CPSF7 in A549, H1299, and HCC827 cells at the protein level compared with that in BEAS‐2B cells (*P* < 0.01, Figure 1b). Among LUAD cell lines, the A549 cell line with relatively high expression of *CPSF7* was selected for subsequent assays (*P* < 0.01, Figure 1a and b).

### 3.2 *CPSF7* acts as an oncogene in LUAD cells

**Figure 2:**
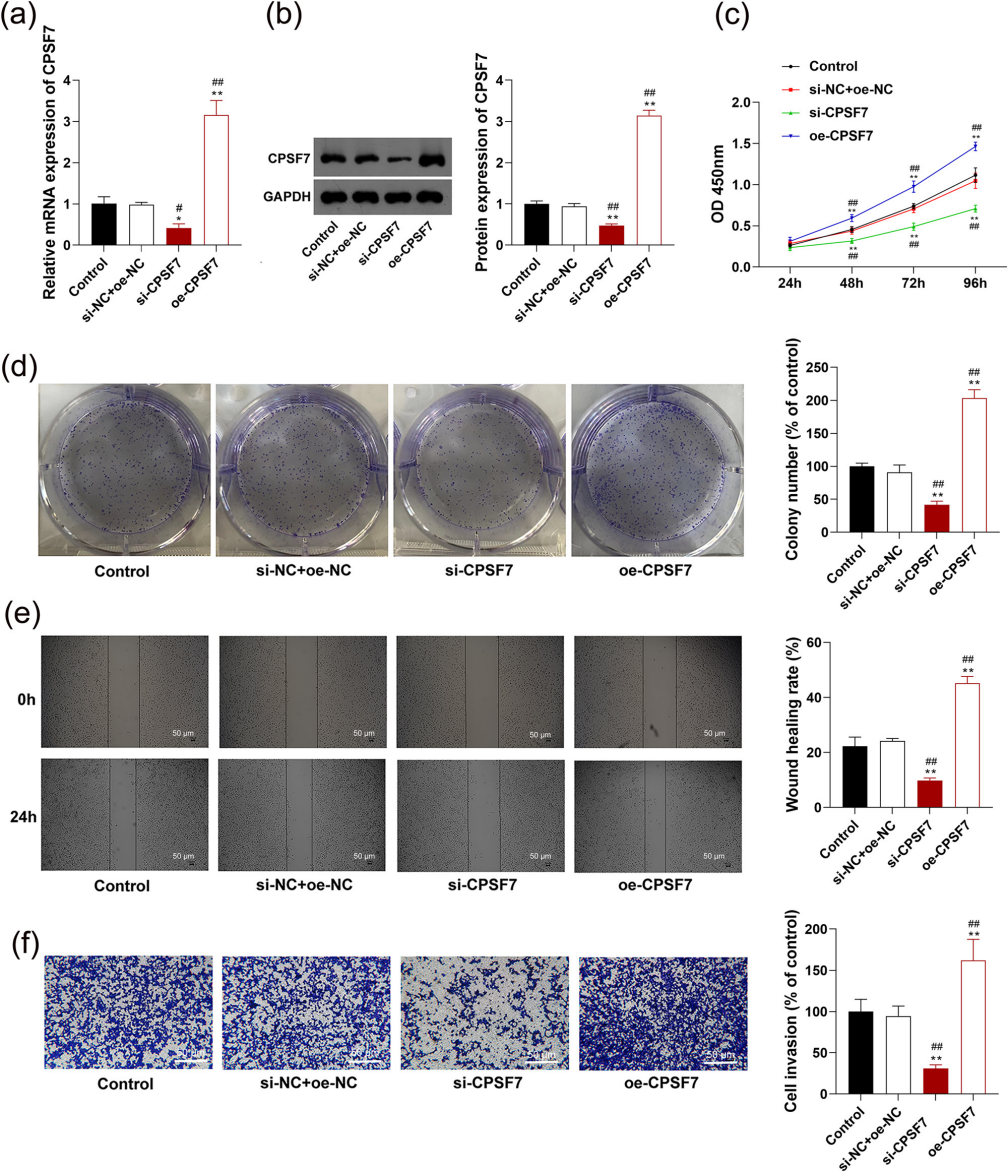
The role of *CPSF7* in the malignant characteristics of LUAD cells: (a) mRNA expression of *CPSF7* was measured using RT-qPCR. (b) Protein expression of CPSF7 was measured by Western blotting. (c) Cell viability was measured by cell counting kit (CCK-8) assay. (d) Colony number was detected by colony formation assay. (e) Cell migration rate was detected by wound healing assay (scale bar = 50 µm). (f) Invasion rate was detected by Transwell assay (scale bar = 50 µm). A549 cells were stably transfected with si-*CPSF7* or oe-*CPSF7*. Each experiment was repeated three times (*n* = 3). ^*^ *P* < 0.05, **P < 0.01 vs Control; ^#^ *P* < 0.05, ^##^ *P* < 0.01 vs si-NC + oe-NC.

**Figure A1:**
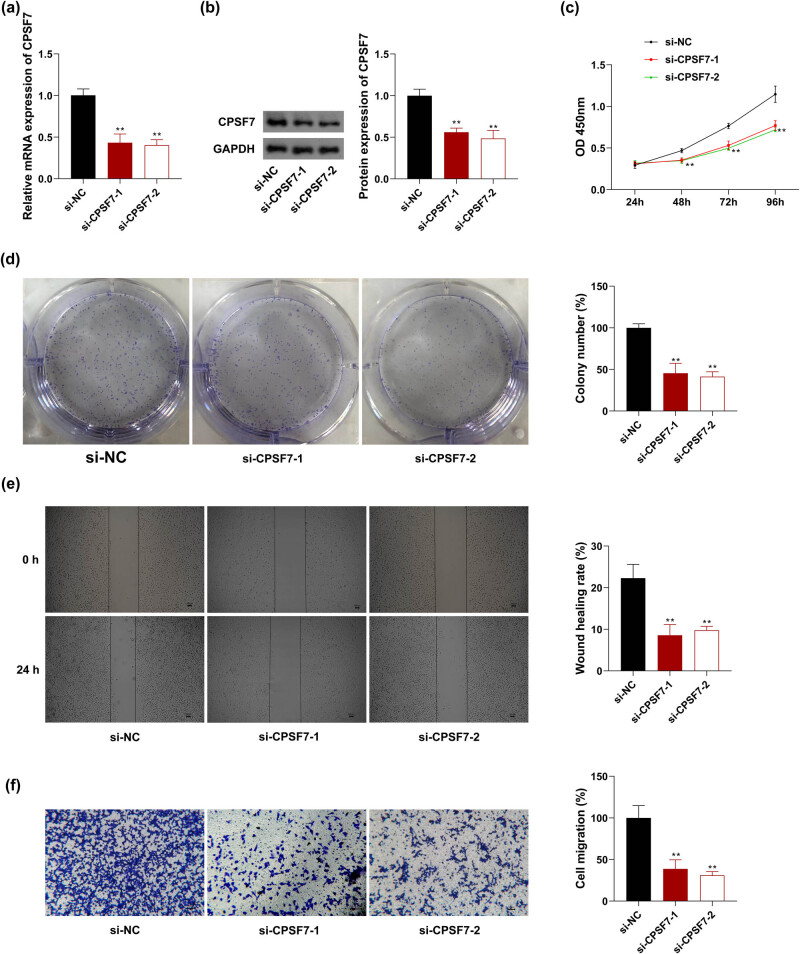
si-CPSF7 was effective in A549 cells. (a) RT-qPCR was used to detect the mRNA expression of CPSF7. (b) Western blotting was used to detect the protein expression of CPSF7. (c) Cell viability detected by CCK-8. (d) Colony number was detected by colony formation assay. (e) Cell migration rate was detected by wound healing assay (scale bar = 50 µm). (f) Invasion rate was detected by Transwell assay (scale bar = 50 µm). A549 cells were stably transfected with si-*CPSF7-1* or si-*CPSF7-2*. Each experiment was repeated 3 times (*n* = 3). ^**^ *P* < 0.01 *vs* si-NC.

*CPSF7* was intervened to explore its role in the malignant characteristics of LUAD cells. As shown in Figure 2a and b, *CPSF7* was significantly downregulated by the transfection of si-*CPSF7* and upregulated by the transfection of oe-*CPSF7* in A549 cells at both the mRNA and protein levels (*P* < 0.05). CCK-8 assay showed that silencing of *CPSF7* significantly decreased the viability of A549 cells at 48, 72, and 96 h post-culturing (*P* < 0.01, Figure 2c). The colony number formed by A549 cells was also significantly decreased by *CPSF7* silencing (*P* < 0.01, Figure 2d). In addition, silencing of *CPSF7* could inhibit the migration and invasion of A549 cells (*P* < 0.01, Figure 2e and f). A549 cells transfected with oe-*CPSF7* exhibited opposite results to those transfected with si-*CPSF7*, presenting enhanced cell viability, colony formation, and cell migration and invasion (*P* < 0.01, Figure 2c–f). Another siRNA targeting CPSF7 to repeat these experiments. Results confirmed the effectiveness of transfected si-CPSF7 (Figure A1).

### 3.3 *CPSF7* activates the AKT/mTOR signaling pathway in LUAD cells

**Figure 3:**
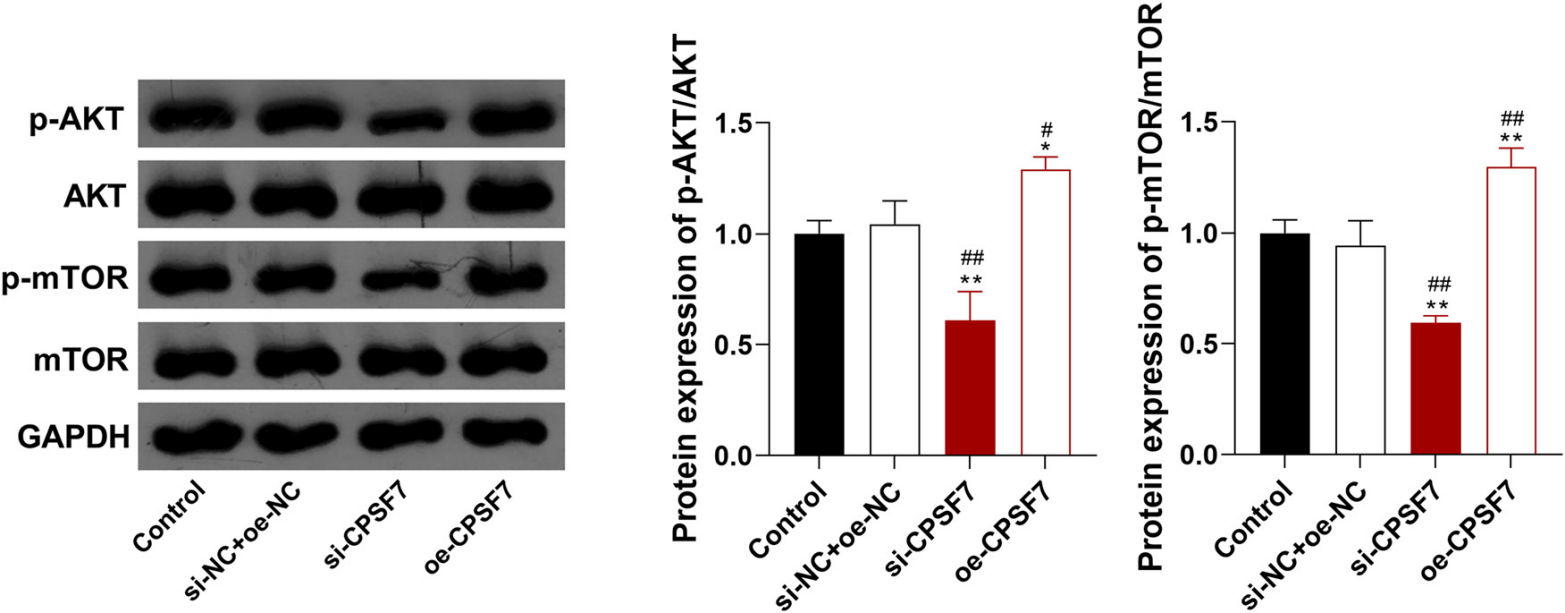
The regulatory role of *CPSF7* on the AKT/mTOR signaling pathway in LUAD cells. A549 cells were stably transfected with si-*CPSF7* or oe-*CPSF7*. The protein expression of p-AKT/AKT and p-mTOR/mTOR was detected by Western blotting. Each experiment was repeated 3 times (*n* = 3). ^*^ *P* < 0.05, ^**^ *P* < 0.01 vs Control; ^#^ *P* < 0.05, ^##^ *P* < 0.01 vs si-NC + oe-NC.

Since the AKT/mTOR signaling pathway plays an important role in tumor progression, the regulatory role of *CPSF7* on this pathway was analyzed. As shown in Figure 3, the transfection of si-*CPSF7* significantly decreased the protein expression of p-AKT/AKT and p-mTOR/mTOR in A549 cells (*P* < 0.01). On the contrary, the transfection of oe-*CPSF7* enhanced the protein expression of p-AKT/AKT and p-mTOR/mTOR in A549 cells (*P* < 0.05).

### 3.4 Silencing of *CPSF7* inhibits the progression of LUAD cells by blocking the AKT/mTOR signaling pathway

**Figure 4:**
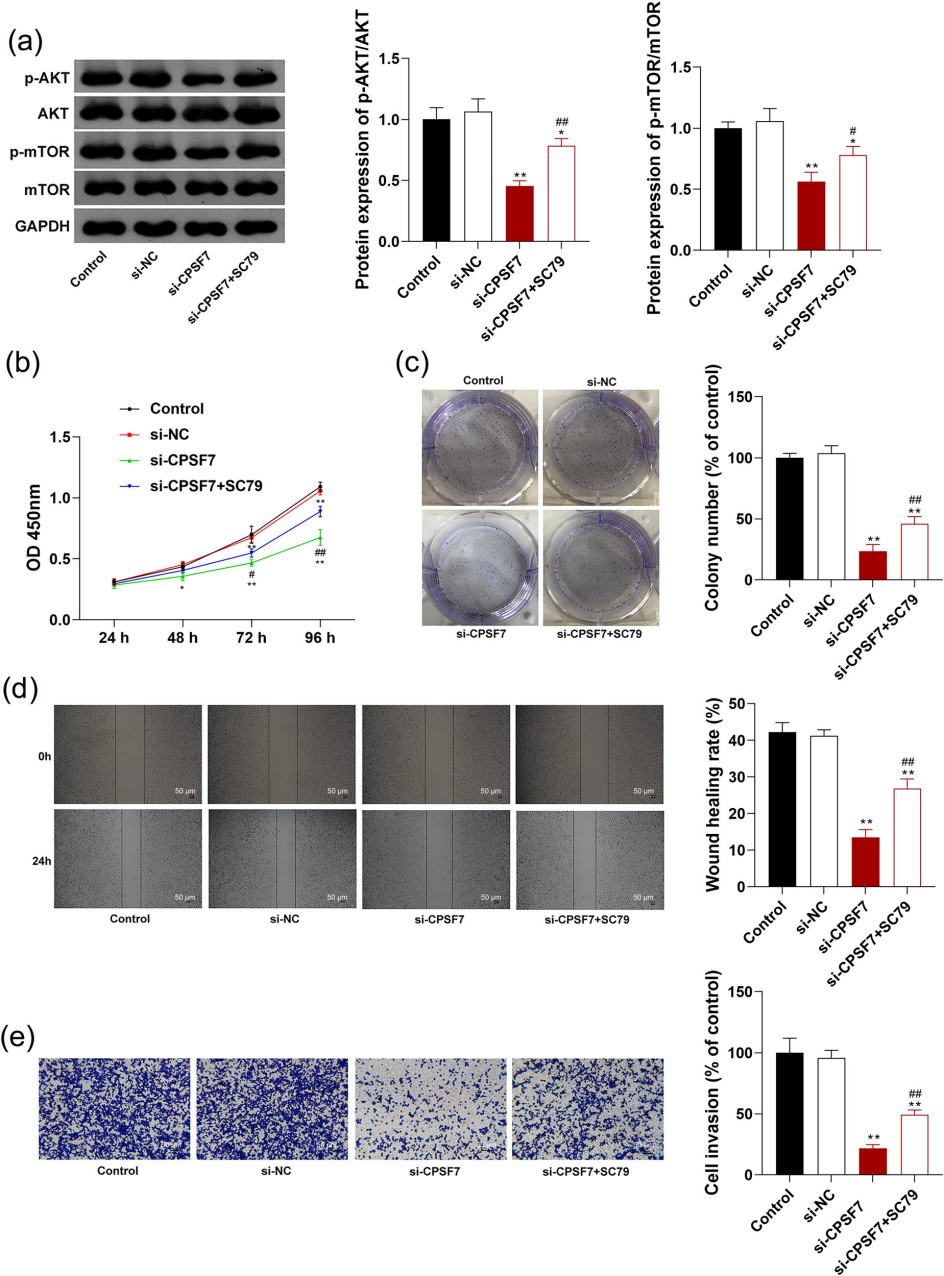
Silencing of *CPSF7* inhibits the malignant characteristics of LUAD cells by blocking the AKT/mTOR signaling pathway. (a) Protein expression of p-AKT/AKT and p-mTOR/mTOR was detected by Western blotting. (b) Cell viability was measured by CCK-8 assay. (c) Colony number was detected by colony formation assay. (d) Cell migration rate was detected by wound healing assay (scale bar = 50 µm). (e) Invasion rate was detected by Transwell assay (scale bar = 50 µm). A549 cells were transfected with si-*CPSF7* and treated with SC79 (an activator of the AKT/mTOR pathway). Each experiment was repeated three times (*n* = 3). ^*^ *P* < 0.05, ^**^ *P* < 0.01 vs Control; ^#^ *P* < 0.05; ^##^ *P* < 0.01 vs si-*CPSF7*.

To further verify whether the antitumor effects of *CPSF7* silencing on LUAD are associated with the blocking of the AKT/mTOR pathway, an activator of the AKT/mTOR pathway SC79 was used. As shown in Figure 4a, the down-regulated p-AKT and p-mTOR in A549 cells transfected with si-*CPSF7* were recovered by the intervention of SC79 (*P* < 0.05). The intervention of SC79 significantly weakened the effects of *CPSF7* silencing on inhibiting the viability and colony formation of A549 cells (*P* < 0.05, Figure 4b and c). In addition, the inhibition of the migration and invasion of A549 cells induced by si-*CPSF7* was also partially eliminated by the intervention of SC79 (*P* < 0.01, Figure 4d and e).

## 4 Discussion

LUAD is the most common type of lung cancer accompanied by high morbidity and mortality worldwide [18]. Nowadays, the comprehensive understanding of the molecular characteristics of lung cancer greatly promotes the development of potential therapeutic targets [19]. In this study, *CPSF7*, a key complex in polyadenylation, was found to be upregulated in LUAD cells. Silencing of *CPSF7* inhibited the malignant characteristics of LUAD cells, presenting a promising therapeutic target. In addition, the antitumor effects of *CPSF7* silencing were closely associated with the inhibiting of the AKT/mTOR signaling pathway.

APA, occurring in over 60% of human genes, has been widely recognized as a key regulatory process of gene expression through generating distinct mRNA 3′ UTR isoforms with different stabilities, translation efficiencies, subcellular localization, and functions [20]. The dysregulation of APA can lead to the imbalance of the cell cycle, contributing to cancer occurrence and progression [21]. As an important component of APA, *CPSF* is usually upregulated in cancers, such as *CPSF1* in HCC and ovarian cancer [8,22], *CPSF3* in HCC [17], *CPSF4* in colorectal and lung cancers [9,10], and *CPSF7* in HCC [12]. In this study, the expression of *CPSF7* was also found to be upregulated in LUAD cells at both mRNA and protein levels. This result is consistent with previous studies and indicates that *CPSF7* may be an oncogene in LUAD. The function of *CPSF7* in LUAD was further analyzed at the cellular level. The results showed that silencing of *CPSF7* inhibited the viability, colony formation, migration, and invasion of LUAD cells. On the contrary, overexpression of *CPSF7* enhanced the malignant characteristics of LUAD cells. These findings illustrate that *CPSF7* acts as an oncogene to promote the progression of LUAD. The antitumor role of *CPSF7* silencing in LUAD is just consistent with that of other CPSFs. For example, the knockdown of *CPSF4* inhibits the proliferation, migration, and invasion of colorectal and lung cancer cells [9,10]. The proliferation and migration of HCC cells are inhibited by the knockdown of *CPSF1*, *CPSF3*, and *CPSF7* [8,12,17]. Combined with the crucial role of *CPSF7* in APA, we suspect that *CPSF7* may drive tumorigenesis by influencing the coding sequence or the 3′-untranslated region of diverse genes. Above all, silencing *CPSF 7* is a promising therapeutic strategy for LUAD.

PI3K/AKT/mTOR pathway is well-known as a crucial intracellular signaling pathway in tumorigenesis, and its activation is closely associated with the malignant hallmarks of cancer cells [15]. The inhibition of the PI3K/AKT/mTOR pathway represents an attractive target for cancer treatments, and massive potential targeted drugs are in preclinical development or early clinical trials [23,24]. In recent years, emerging genes have been revealed to be the potential therapeutic targets of lung cancer by inhibiting the PI3K/AKT/mTOR pathway, such as *SREBP* [25], *DOK7V1* [26], *HRH3* [27], *SLFN5* [28], and *FABP5* [29]. In addition, the antitumor potential of some CPSFs is also mediated by the PI3K/AKT pathway. For example, *CPSF4* knockdown inhibits the PI3K/AKT pathway in LUAD [10]; *CPSF3* knockdown inhibits the PI3K/AKT pathway in HCC [17]; *CPSF7* knockdown inhibits the PTEN/AKT pathway in HCC [12]. In this study, the potential action mechanisms of *CPSF 7* involving the AKT/mTOR pathway were further analyzed in LUAD. Similar to previous studies mentioned above, *CPSF7* knockdown inhibited the AKT/mTOR pathway in LUAD cells. We suspect that the inhibition of the AKT/mTOR pathway may contribute to the anti-tumor effects of *CPSF7*. Encouragingly, the following feedback experiments showed that SC79 significantly weakened the effects of *CPSF7* on inhibiting the viability, colony formation, migration, and invasion of LUAD cells. Therefore, we conclude that silencing of *CPSF7* inhibits the malignant characteristics of LUAD cells by blocking the AKT/mTOR signaling pathway.

## 5 Conclusions

In conclusion, *CPSF7* is upregulated in LUAD cells. Silencing of *CPSF7* inhibits the viability, colony formation, migration, and invasion of LUAD cells by blocking the AKT/mTOR signaling pathway. These findings indicate that *CPSF7* may be a promising therapeutic target for LUAD. However, this study is limited to the cellular level. Further research on in-depth mechanisms involving *CPSF7* is still needed.
